# Von Willebrand Factor and ADAMTS-13 Are Associated with the Severity of COVID-19 Disease

**DOI:** 10.3390/jcm11144006

**Published:** 2022-07-11

**Authors:** Nataliya Dolgushina, Elena Gorodnova, Olga Beznoshenco, Andrey Romanov, Irina Menzhinskaya, Lyubov Krechetova, Gennady Sukhikh

**Affiliations:** National Medical Research Center for Obstetrics, Gynecology and Perinatology Named after Academician V.I. Kulakov of Ministry of Healthcare of Russian Federation, 117997 Moscow, Russia; n_dolgushina@oparina4.ru (N.D.); e_gorodnova@oparina4.ru (E.G.); o_beznoshchenko@oparina4.ru (O.B.); i_menzinskaya@oparina4.ru (I.M.); l_krechetova@oparina4.ru (L.K.); g_sukhikh@oparina4.ru (G.S.)

**Keywords:** COVID-19, von Willebrand factor, ADAMTS-13, hypercoagulation, thrombosis

## Abstract

Coagulopathy in COVID-19 patients is presumably based on systemic hypercoagulation with the inflammatory response. As a result of endothelial dysfunction, tissue factor and von Willebrand factor (vWF) are released into the blood stream, which leads to prothrombinase activation. The vWF/ADAMTS-13 ratio can be used for monitoring the severity of the disease. This observational prospective study included 141 patients with COVID-19. In patients with mild COVID-19 (group 1), the assessment was performed on the 3rd–7th day of illness (point 1) and 14–28 days after recovery (point 2). In patients with moderate (groups 2) and severe (group 3) COVID-19, the assessment was performed during hospitalization (point 1) and after 14 days (point 2). The vWF:RCo/ADAMTS-13:activity (point 1), vWF/ADAMTS-13 (point 2) and vWF:RCo/ADAMTS-13:activity (point 2) ratios were significantly higher in patients with moderate and severe COVID-19. Moreover, in these patients, both ratios increased after recovery (point 2), which is a negative prognostic factor of thrombotic complications. Thus, COVID-19 is characterized by a decrease in the concentration and activity of ADAMTS-13 metalloproteinase, especially in patients with the severe form of COVID-19. A decrease in ADAMTS-13 activity results in an increase in vWF concentration and activity so the ratio of vWF to ADAMTS-13 changes significantly.

## 1. Introduction

Information about the epidemiology, clinical features, prevention and treatment of COVID-19 is still limited and controversial [1]. Coagulopathy in COVID-19 patients is presumably based on systemic hypercoagulation, which rises with the inflammatory and autoimmune responses [2,3]. Hyperactivation of plasma coagulation is registered in patients with COVID-19 and leads to the formation of pathological fibrin and blood clots in the vessels and microvasculature of the lungs [4]. However, endothelial dysfunction also can play a role in the increased coagulation [5].

As a result of endothelial cell dysfunction, tissue factor and von Willebrand factor (vWF) are released into the blood stream, which leads to prothrombinase activation by coagulation factor VII. In turn, increased vWF level leads to platelet adhesion to the collagen substrate (collagen type I and III) at the sites of endothelial damage through GP1b, which is expressed on the surface of platelets. Where there is thrombosis exocytosis of dense granules containing low-molecular-weight compounds such as ADP, ATP, serotonin, calcium and magnesium ions, GDP, GTP, etc. and alpha granules containing vWF, fibrinogen, fibronectin, thrombospondin, coagulation factors, fibrinolysis and anticoagulants (plasminogen, protein S), pro-inflammatory cytokines and chemokines (platelet factor 4, ß-thromboglobulin) occurs. All this leads to platelet autoactivation and changes in the expression profile of their surface receptors.

ADAMTS-13 is a metalloprotease enzyme required for vWF cleavage [6,7]. Decrease in ADAMTS-13 activity leads to incomplete cleavage of extra-large vWF multimers produced by the endothelium, which leads to spontaneous platelet aggregation in the microvasculature of some organs. This is followed by the formation of microthromboses, microangiopathic hemolytic anemia with thrombocytopenia (usually severe) and multisystem organ damage. The brain, heart and kidneys are most commonly affected [8]. There is a strong relationship between endothelial injury, inflammation, the activation of the coagulation cascade and the severity of COVID-19. The association between reduced ADAMTS-13 activity and COVID-19 mortality has been proven in multiple studies [9,10]. ADAMTS-13 at hospital admission plays a significant role in assessing the risk of death in patients with COVID-19 [11]. In patients with sepsis, low levels of ADAMTS-13 correlate with high levels of von Willebrand factor and poor prognosis [12]. According to the cohort study data obtained by Tiscia G. et al., a vWF/ADAMTS-13 ratio above 6.5 is associated with mortality in COVID-19 patients. A ratio above 5.7 (at the time of hospitalization) is associated with admission to the intensive care unit. Additionally, the vWF/ADAMTS-13 ratio correlates with C-reactive protein and D-dimer levels [13]. Thus, the analysis of the vWF/ADAMTS-13 ratio makes it possible to predict the course of COVID-19 when a patient is admitted to a hospital and can also be a useful tool for monitoring the severity of the disease.

## 2. Materials and Methods

The observational prospective study included 141 patients with COVID-19:

Group 1—patients with mild COVID-19 (*n* = 39).

Group 2—patients with moderate COVID-19 (*n* = 65).

Group 3—patients with severe COVID-19 (*n* = 37).

The severity of COVID-19 was established on the basis of the interim guidelines of the Ministry of Health of the Russian Federation “Prevention, diagnosis and treatment of a new coronavirus infection (COVID-19)”:

- Mild COVID-19:
  - body temperature < 38 °C, cough, weakness, sore throat;
  - absence of criteria for moderate and severe COVID-19.
- Moderate COVID-19:
  - body temperature > 38 °C;
  - respiratory rate > 22 per minute;
  - shortness of breath during physical exertion;
  - changes in CT (radiography), typical of a viral lesion;
  - SpO2 < 95%;
  - serum CRP > 10 mg/L.
- Severe COVID-19:
  - respiratory rate > 30 per minute;
  - SpO2 ≤ 93%;
  - PaO2/FiO2 ≤ 300 mmHg;
  - decreased level of consciousness, agitation;
  - unstable hemodynamics (systolic blood pressure less than 90 mm Hg or diastolic blood pressure less than 60 mm Hg, diuresis less than 20 mL/hour)
  - arterial blood lactate > 2 mmol/l;
  - qSOFA > 2 points.

The study was carried out at:

1. National Medical Research Center for Obstetrics, Gynecology and Perinatology, named after Academician V.I.Kulakov of the Ministry of Healthcare of the Russian Federation, Moscow, Russia
2. F.I. Inozemtsev City Clinical Hospital, Moscow, Russia
3. A database containing information on 18 patients with a severe form of COVID-19 complicated by a feasibility study that was provided by Ataullakhanov Fazoil Inoyatovich (CTP FHF RAS, research work “Use of the Thrombodynamics test in COVID-19: identification of early predictors of the development of severe pneumonia and development of effective measures for its prevention”, registration number: AAAA-A20-120111090014-6) [14].

Inclusion criteria:

- Age over 18 years.
- Signed informed consent.

Non-inclusion criteria:

- For women: pregnancy or lactation.
- Hereditary deficiency of blood coagulation factors predisposing to hemorrhagic conditions.
- Purpura and other hemorrhagic conditions.
- Oncological diseases for the period of illness.
- Transplanted organs.
- HIV infection.
- Syphilis.
- Other acute infectious diseases.
- Continuous use of anticoagulants/antiplatelet agents.

Exclusion Criteria:

- The need for surgery during COVID-19.
- Patient refusal to continue participation in the study.

In patients with mild COVID-19 (group 1), the assessment was performed twice. Point 1 was on the 3rd–7th day of illness and point 2 at 14–28 days after recovery. In patients with moderate (groups 2) and severe (group 3) COVID-19, the assessment was performed twice. Point 1 was during hospitalization and point 2 was 14 days after point 1. No acute thrombosis was recorded during the study. There were no deaths, and all patients were discharged from the hospital in a satisfactory condition.

The ADAMTS-13 blood plasma concentration and activity were determined using ELISA kits “TECHNOZYM ADAMTS-13 Antigen” and “TECHNOZYM ADAMTS-13 Activity” (Technoclone GmbH, Vienna, Austria). The ADAMTS-13 inhibitor was determined using ELISA kit «TECHNOZYM ADAMTS-13 Inhibitor» (Technoclone GmbH, Vienna, Austria). The assessment was carried out according to the manufacturer’s instructions.

The von Willebrand factor (vWF) antigen and ristocetin-cofactor activity (vWF:RCo) assessment was performed using automatic coagulometer ACL TOP 700 (Instrumentation Laboratory, Lexington, KY, USA).

Statistical data processing was performed using Microsoft Excel and Statistica V10 (USA). Risks (%) were calculated to evaluate qualitative data. The χ2 test was used to compare categorical data in two or more groups. The type of quantitative data distribution was determined by Kolmogorov–Smirnov test and visual data analysis. Mean values with a standard deviation (M ± SD) were calculated for the data with normal distributions and parametric statistics was used (ANOVA in 3 groups and pared *t*-test in 2 groups). Differences were considered statistically significant at *p* < 0.05.

Ethical approval was obtained from the local ethics committee.

## 3. Results

The characteristics of the patients (gender, age, height, weight and BMI) are presented in Table 1. Male gender, old age and high BMI were associated with the severity of COVID-19.

It is assumed that the imbalance between vWF and ADAMTS-13 in COVID-19, including a decrease in the level and activity of ADAMTS-13, may create microthrombotic conditions leading to secondary thrombotic microangiopathy. Insufficiency of ADAMTS-13 leads to the accumulation of very large vWF multimers, while the spontaneous formation of microthrombi in arterioles and capillaries is induced, followed by the development of ischemia. Deficiency in ADAMTS-13 can trigger an acute episode of TTP. In addition to the genetic form of TTP (Upshaw–Schulman syndrome), an acquired form is also isolated, due to the presence of antibodies to ADAMTS-13 or its inhibitor. Therefore, when studying the functional activity of ADAMTS-13, not only the level of antigen and activity of ADAMTS-13 in the blood was determined but also the presence of an inhibitor of ADAMTS-13.

At point 1, the ADAMTS-13 antigen level decreased with the severity of the disease, but it was not statistically significant. At point 2, the ADAMTS-13 antigen level did not differ between groups 1 and 2, but it was significantly lower in patients with severe COVID-19. Additionally, ADAMTS-13 antigen blood concentration decreased at the 2nd point in comparison with the 1st point only in this group of patients (*p* = 0.0002).

At point 1, ADAMTS-13 activity in moderate and severe COVID-19 was significantly lower than in mild COVID-19. At point 2, ADAMTS-13 activity decreased as the COVID-19 severity increased. At the same time, the activity of ADAMTS-13 in the second point compared with the first point did not differ significantly between groups.

At point 1, the level of ADAMTS-13 inhibitor did not differ significantly between groups, although it was slightly higher in patients with severe COVID-19. At point 2, the level of ADAMTS-13 inhibitor was significantly lower in patients with moderate COVID-19. At the same time, there was an increase in ADAMTS-13 inhibitor in patients with mild COVID-19 (Table 2).

In moderate and severe COVID-19, low ADAMTS-13 levels (below referral) were detected most often (Table 3).

We analyzed the association between the level and activity of ADAMTS-13 and the vWF blood level. A significant ADAMTS-13 blood level decrease in patients with severe COVID-19 was accompanied by a significant increase in the vWF:RCo blood level from point 1 to point 2 (Table 4). So, even during the period of convalescence, the prothrombotic activity of the vascular endothelium took place.

**Figure 1:**
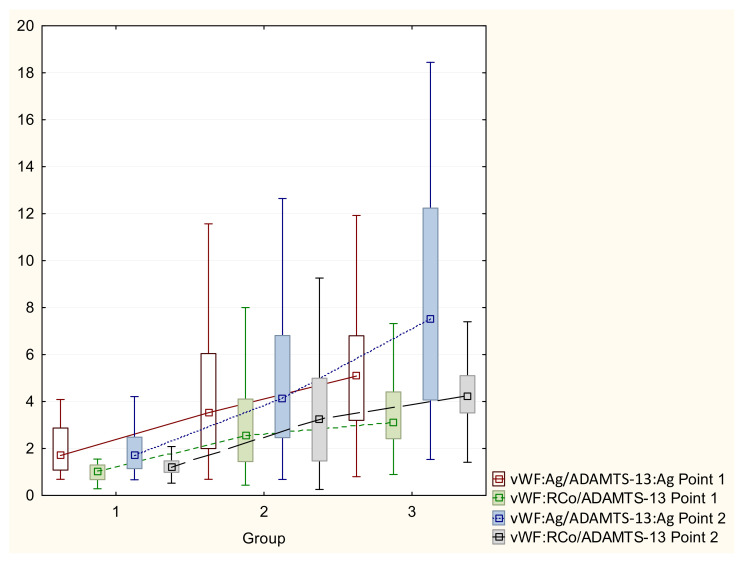
vWF/ADAMTS-13 ratio in patients with COVID-19.

The vWF to ADAMTS-13 ratio in patients with COVID-19 is shown in Table 4 and Figure 1. To calculate these ratios, the ADAMTS-13 concentration and vWF activity were measured in IU/mL (%/100). The literature contains data on the prognostic value of these ratios in terms of the severity of infection [13]. For example, a vWF:RCo/ADAMTS-13:activity level > 5.7 is associated with ICU admission, and a level > 6.5 was associated with increased patient mortality.

The vWF:RCo/ADAMTS-13:activity (Point 1), vWF/ADAMTS-13 (Point 2) and vWF:RCo/ADAMTS-13:activity (Point 2) ratios were significantly higher in patients with moderate and severe COVID-19. Moreover, in these patients, both ratios increased after recovery (Point 2), which is a negative prognostic factor of thrombotic complications.

Using ROC analysis, we found the threshold values for these indicators to distinguish patients with mild COVID-19 from patients with moderate and severe COVID-19 (Table 5).

## 4. Discussion

In this study, we looked closely at the role of vWF and ADAMTS-13 in COVID-19. We have proven that a decrease in the level and activity of ADAMTS-13 in patients with severe COVID-19 can lead to an imbalance between vWF and ADAMTS-13. This imbalance can be caused by several factors. ADAMTS-13 is a metalloprotease enzyme required for vWF cleavage [6,7]. This means that these two factors are inversely related. Thus, a decrease in ADAMTS-13 activity leads to an increase in the level of vWF. On the other hand, vWF are released into the blood stream due to the endothelial cell dysfunction caused by COVID-19 [5]. According to our data, both of these mechanisms are more pronounced in severe forms of COVID-19. These mechanisms are parts of a system with complex regulatory mechanisms, a more detailed study of which may be the goal of further research. The presence of antibodies to ADAMTS-13 may also contribute to the decrease in ADAMTS-13 activity in patients with COVID-19 of varying severity. This leads to the activation of prothrombinase by the coagulation factor VII and the beginning of the coagulation cascade, which was described earlier. Thus, we have confirmed a relationship between endothelial injury, inflammation, activation of the coagulation cascade and the severity of COVID-19. The revealed changes increase with the course of the disease.

The diagnostic and prognostic value of vWF/ADAMTS-13 ratio has been studied in several studies [12,13]. In our study we found that, at the time of patient’s admission to a hospital, the vWF:RCo/ADAMTS-13:activity ratio has a greater diagnostic value than the vWF/ADAMTS-13 ratio. The threshold levels for these indicators were 1.55 and 2.45, respectively. These levels can be used for additional early assessment of the severity of COVID-19.

In the light of the presented data, it seems promising to further study the role of vWF and ADAMTS-13 in the development of complications of COVID-19 as well as to study their relationship with the long-term outcomes of this disease.

## 5. Conclusions

Thus, COVID-19 is characterized by a decrease in the concentration and activity of ADAMTS-13 metalloproteinase, especially in patients with the severe form of COVID-19. A decrease in ADAMTS-13 activity results in an increase in vWF concentration and activity, so the ratio of vWF to ADAMTS-13 changes significantly. The presence of anti-ADAMTS-13 antibodies may also decrease ADAMTS-13 activity in COVID-19 patients.

## Figures and Tables

**Table 1:** Characteristics of patients.

Characteristics	MILD (*n* = 39)	MODERATE (*n* = 65)	SEVERE (*n* = 37)	*p*-Value
Gender male	7 (17.9%)	24 (36.9%)	22 (59.5%)	0.0009 ***
Gender female	32 (82.1%)	41 (63.1%)	15 (40.5%)	
Age, years	38 (34–54)	60 (43–78)	63 (53–71)	0.0001 **
Height, m	1.67 ± 0.09	1.68 ± 0.08	1.69 ± 0.07	0.5664 *
Body weight, kg	71.3 ± 15.4	78.5 ± 20.3	83.3 ± 12.5	0.0094 *
BMI, kg/m^2^	25.2 ± 4.4	27.4 ± 6.2	29.1 ± 5.3	0.0092 *
Smoking	4 (10.3%)	10 (15.4%)	1 (2.7%)	0.1355 ***
Alcohol consumption	13 (33.3%)	12 (18.5%)	9 (24.3%)	

* M ± Sd, ANOVA; ** Me(X–Y), Kruskal–Wallis test; *** *n* (%), ꭓ2-test.

**Table 2:** The blood concentration and activity of ADAMTS-13 and the blood concentration of the ADAMTS-13 inhibitor in patients COVID-19.

Point of Study (Reference)	MILD (*n* = 39)	MODERATE (*n* = 65)	SEVERE (*n* = 37)	*p*-Value
ADAMTS-13 Point 1 (0.41–1.41 mU/mL)	0.83 ± 0.22	0.75 ± 0.26	0.70 ± 0.3	0.1678
				0.1584 *; 0.0662 **;
				0.4461 ***
ADAMTS-13 Point 2 (0.41–1.41 mU/mL)	0.78 ± 0.22	0.73 ± 0.29	0.46 ± 0.23	<0.0001
	*p* = 0.4095 ^	*p* = 0.6458 ^	*p* = 0.0002 ^	0.2952 *; <0.0001 **;
				<0.0001 ***
ADAMTS-13 activity Point 1 (0.4–1.3 mU/mL)	1.02 ± 0.18	0.89 ± 0.20	0.89 ± 0.23	0.0093
				0.0027 *; 0.0116 **;
				0.9720 ***
ADAMTS-13 activity Point 2 (0.4–1.3 mU/mL)	0.95 ± 0.12	0.89 ± 0.15	0.79 ± 0.22	0.0007
	*p* = 0.0533 ^	*p* = 0.9541 ^	*p* = 0.0970 ^	0.0391 *; 0.0005 **;
				0.0228 ***
ADAMTS-13 inhibitor Point 1 (<12 mU/mL)	7.60 ± 3.42	7.51 ± 4.47	9.36 ± 4.49	0.0815
				0.9089 *; 0.0578 **;
				0.0567 ***
ADAMTS-13 inhibitor Point 2 (<12 mU/mL)	10.49 ± 5.26	8.42 ± 4.89	11.91 ± 7.07	0.0134
	*p* = 0.0052 ^	*p* = 0.2763 ^	*p* = 0.0734 ^	0.0479 *; 0.3328 **;
				0.0060 ***

M ± SD, ANOVA; *, **, ***—Tukey test (mild vs. moderate, mild vs. severe, moderate vs. severe); ^ Point 1 vs. Point 2 (paired *t*-test).

**Table 3:** Decreased ADAMTS-13 blood level in patients with moderate and severe COVID-19.

Point of Study (Reference)	MILD (*n* = 39)	MODERATE (*n* = 65)	SEVERE (*n* = 37)	*p*-Value
Point 1	1 (2.6%)	12 (18.5%)	8 (21.6%)	0.0180 *
				0.0110 **
Point 2	3 (7.7%)	10 (15.4%)	18 (48.6%)	0.2500 *
				0.0001 **

Abs. (%), ꭓ2; *, **—(mild vs. moderate, mild vs. severe).

**Table 4:** vWF/ADAMTS-13 ratio in patients with COVID-19.

Point of Study	MILD (*n* = 39)	MODERATE (*n* = 65)	SEVERE (*n* = 37)	*p*-Value
vWF/ADAMTS-13 Point 1	2.7 ± 3.2	5.7 ± 9.7	5.6 ± 3.7	0.1428 0.1184 *; 0.1054 **; 0.2154 ***
vWF:RCo/ADAMTS-13:activity Point 1	1.5 ± 1.8 *p* = 0.0248 ^	3.1 ± 2.1 *p* = 0.0378 ^	3.6 ± 2.2 *p* = 0.0217 ^	0.0005 0.0127 *; 0.0097 **; 0.4478 ***
vWF/ADAMTS-13 Point 2	2.2 ± 1.9	6.9 ± 12.3	10.3 ± 9.9	0.0065 0.0195 *; 0.0054 **; 0.0845 ***
vWF:RCo/ADAMTS-13:activity Point 2	1.5 ± 1.7 *p* = 0.0298 ^	3.5 ± 2.2 *p* = 0.0281 ^	4.5 ± 2.0 *p* = 0.0285 ^	<0.0001 0.0065 *; 0.0019 **; 0.0498 ***

M ± SD, ANOVA; *, **, ***—Tukey test (mild vs. moderate, mild vs. severe, moderate vs. severe); ^ Point 1 vs. Point 2 (paired *t*-test).

**Table 5:** ROC analysis.

Point of Study	Threshold	AUC	Se	Sp
vWF/ADAMTS-13 Point 1	2.45	74.2%	74.5%	70.0%
vWF:RCo/ADAMTS-13:activity Point 1	1.55	82.6%	73.3%	90.0%
vWF/ADAMTS-13 Point 2	2.55	81.7%	78.5%	78.9%
vWF:RCo/ADAMTS-13:activity Point 2	1.55	80.4%	77.1%	78.9%

## Data Availability

The data presented in this study are available on request from the corresponding author. The data are not publicly available due to privacy or ethical restrictions.
